# How digital transformation in “diverse directions” activates enterprise total factor productivity: A mechanism study based on dynamic capability reconfiguration

**DOI:** 10.1371/journal.pone.0347212

**Published:** 2026-04-28

**Authors:** Peng Song, Jiahui Li, Ning Wu

**Affiliations:** Department of Finance and Economics, Jiangsu University, Zhenjiang, Jiangsu, China; Instituto Tecnologico Autonomo de Mexico, MEXICO

## Abstract

Digital transformation is a key force driving high-quality economic development, yet the economic consequences of different digital technology directions vary significantly. Existing research often treats it as a homogeneous whole, overlooking the “diverse direction” characteristics of technologies, making it difficult to explain the “productivity paradox.” From the perspective of dynamic capability reconstruction, this paper constructs a theoretical model of “Digital ‘Diverse Direction’ Transformation—Dynamic Capability—Total Factor Productivity (TFP).” Using the LDA topic model to conduct semantic analysis on the annual reports of A-share listed companies, we categorize digital transformation into five directions: Artificial Intelligence (AI), Big Data, Cloud Computing, Blockchain, and Digital Technology Application. We empirically test their differentiated impacts on enterprise TFP and the underlying mechanisms. The findings are as follows: First, while digital transformation overall significantly promotes enterprise TFP, there is significant “technological heterogeneity” among different directions. AI has the strongest promoting effect, followed by Big Data and Digital Technology Application, whereas the impacts of Cloud Computing and Blockchain are not yet significant. This provides micro-evidence for explaining the “productivity paradox.” Second, mechanism tests indicate that dynamic capability reconstruction is the key transmission path; digital transformation enhances TFP by strengthening organizational coordination and integration capabilities, change and reconstruction capabilities, and learning and absorption capabilities. Third, heterogeneity analysis reveals that digital transformation exerts a more pronounced effect on enhancing productivity in labor-intensive and technology-intensive industries, as well as in the manufacturing sector. These conclusions deepen the theoretical understanding of the economic consequences of digital transformation and provide empirical evidence and decision-making references for enterprises to choose adaptable and differentiated transformation paths.

## Introduction

In an era where the wave of the digital economy sweeps across the globe, digital transformation has become a key engine for enterprises to break through traditional development bottlenecks and reshape core competitiveness. The deep penetration of digital technologies such as Artificial Intelligence, Big Data, and Cloud Computing has led to “diverse direction” characteristics in the technical orientation of digital transformation, driving multi-dimensional improvements in dynamic capabilities regarding resource integration, technology R&D, and organizational management. According to the “Global Digital Economy Development Research Report,” global investment in digital transformation is expected to reach $3 trillion by 2025, with a five-year compound annual growth rate of approximately 15.4% from 2023 to 2028, becoming a key force driving global economic transformation and upgrading. As a major player in the digital economy, China’s digital economy scale reached 53.9 trillion yuan in 2023, contributing 66.45% to GDP growth. Thus, under the premise of maintaining high-speed growth in scale, China’s digital economy has gradually become an important component of the national economy, effectively supporting stable economic growth.

However, in practice, the effects of transformation are significantly divergent: some enterprises achieve efficiency leaps, while others fall into an “efficiency trap” [1]. Theoretically, there is still controversy over whether digitization enhances Total Factor Productivity (TFP). Most scholars hold a positive view, believing it can reduce costs, increase efficiency, and promote innovation [2,3], playing active roles in technological innovation [4], financing constraints [5], and business model innovation [6]. Yet, the “productivity paradox” persists: [7] found that not all enterprises benefit; [8] confirmed that while transformation expands scale, it does not necessarily improve TFP; [9,10] found an inverted U-shaped nonlinear relationship between the two. This indicates that the actual effect of digital transformation is not always ideal.

Although “digital transformation” has become a core strategic issue for enterprises, its technical connotation and implementation paths are not homogeneous but exhibit significant “diverse direction” characteristics. This difference is first reflected at the macro-industrial level: in 2023, the combined revenue of cloud services and big data services in China’s cloud computing sector reached 1.247 trillion yuan, while the core industry scale of Artificial Intelligence was approximately 580 billion yuan (“China Digital Economy Development Research Report (2024)”). The obvious gap in economic scale reflects a structural differentiation in the commercial maturity and market penetration depth of different digital technologies. More importantly, this macro-level difference has transmitted to micro-enterprise practices. [11], based on text analysis of listed company annual reports, found that the adoption rates of various digital technologies within enterprises are highly uneven, with Cloud Computing and Big Data having significantly higher penetration rates than Blockchain technology. This means that even under the same wave of “digital transformation,” the technology portfolios, investment focuses, and strategic goals relied upon by enterprises may be vastly different. If research simply aggregates these significantly different technologies into a vague “digital index,” it easily obscures the true mechanisms by which different types of digital technologies affect corporate performance.

Upon closer examination, existing research mostly treats digital transformation as a homogeneous whole, neglecting the “diverse direction” characteristics of technology directions. Macroscopically, different digital technologies (such as Cloud Computing and AI) show structural differentiation in economic scale; microscopically, enterprise adoption rates are highly uneven [11]. Simply aggregating them into a general index easily blurs the real mechanisms of different technologies on performance. In fact, the action paths of different technologies vary significantly: AI promotes industrial chain coupling through “intelligent manufacturing integration” [12], while Big Data mainly improves TFP by reducing costs, optimizing management, and promoting green innovation [13]. Without adaptable dynamic capabilities, this could instead lead to resource misallocation. Therefore, the “diverse direction” characteristic may be a new perspective for explaining the “productivity paradox.”

Existing literature still has limitations in granularity and mechanistic explanatory power: First, ignoring technological heterogeneity can easily lead to an “average number trap,” with few studies systematically testing the differentiated impacts and time-lag characteristics of different technology directions. Second, measurement methods struggle to capture deep semantics and lack a refined perspective of “dynamic capability reconstruction.” Third, against the background of high-quality development, clarifying which technology directions take effect immediately and which have lags has urgent practical significance for enterprises to avoid traps.

Based on this, this paper attempts to address: First, whether the various technology directions of enterprise digital “diverse direction” transformation have improved TFP; Second, whether there are differences in their enhancement mechanisms? This paper constructs a theoretical model of “Digital ‘Diverse Direction’ Transformation—Dynamic Capability—Total Factor Productivity,” uses the LDA topic model to subdivide technology directions, and deconstructs dynamic capabilities into three dimensions: organizational coordination and integration, change and reconstruction, and learning and absorption, to empirically test differentiated impacts and mechanisms.

The marginal contributions of this paper are mainly reflected in: First, perspective innovation: for the first time, it systematically deconstructs digital transformation into five directions: Artificial Intelligence, Big Data, Cloud Computing, Blockchain, and Digital Technology Application, finding significant “time heterogeneity” in their impacts, providing new evidence to explain the “productivity paradox.” Second, methodological innovation: using the LDA topic model to optimize the measurement system, overcoming the defects of traditional word frequency statistics. Third, theoretical deepening: analyzing how different technology directions differentially reshape the three core dynamic capabilities, opening the “black box” of how transformation affects productivity, and providing theoretical support for enterprises to formulate differentiated transformation strategies.

### Digital transformation and enterprise total factor productivity

Digital transformation enhances enterprise Total Factor Productivity (TFP) through three major mechanisms. First, regarding the organizational coordination and integration effect, digital applications enhance the intensity of information disclosure, build financing platforms to alleviate financing constraints [5], and guide investor attention by releasing positive signals, thereby improving stock liquidity [10]. Second, regarding the technological innovation effect, digitization strengthens enterprise connections and network integration, accelerating knowledge spillovers [14]; it constructs unified supply chain platforms to achieve efficient collaboration, optimize resource allocation, and improve downstream productivity [15]; simultaneously, it enhances information analysis capabilities, aiding in the excavation and conversion of potential consumer demands [16]. Finally, regarding the management efficiency effect, digitization reduces internal communication and information acquisition costs, enhancing hierarchical synergy [17]; it promotes the evolution of production organization towards flattening and automation, precisely allocating labor, systematically reducing costs, and improving efficiency and quality stability [18]. In summary, digital transformation has a significant positive impact on TFP.

However, the application effects of different digital technologies vary significantly. [3] and [19] analyzed the impact of transformation on financial performance from an overall perspective; [20] and [21] focused on the specific impacts of Blockchain and Artificial Intelligence, respectively. [11] found that except for Blockchain, all other types of technologies can significantly improve Return on Total Assets; [22] pointed out that the integrated application of different technologies has a “1+1>2” multiplier effect on TFP; [23] also confirmed that except for Blockchain, the enhancement effects of other technology dimensions on high-quality development are significant. Based on this, we propose the hypothesis:

- H1: Digital transformation has a significant positive impact on enterprise Total Factor Productivity.
- H1a: Different digital technology directions have different effects on enterprise Total Factor Productivity.

### The mediating role of dynamic capabilities

[24] and [25] defined dynamic capabilities as the ability of an enterprise to integrate, build, and reconfigure internal and external resources to cope with environmental changes. Subsequent scholars [26,27] further emphasized their behavioral orientation in innovating competitive advantages and reallocating resources. Comprehensively, dynamic capabilities are the core abilities for an enterprise to identify and allocate resources, improve efficiency through technological innovation, and optimize management to support long-term development.

Regarding their dimensions, although there are various classifications in academia, [25] proposed three dimensions: sensing opportunities, seizing opportunities, and transforming/reconfiguring; [27] defined them as adaptive, absorptive, and innovative capacities; [28] constructed a four-dimensional system of environmental insight, change renewal, technological flexibility, and organizational flexibility; [29] divided them into a five-dimensional framework of environmental adaptation, organizational change, resource integration, learning, and strategic isolation mechanisms. However, the core essentially boils down to resources, change/innovation, and learning. Given that existing research generally adopts the three-dimensional framework of “Organizational Coordination and Integration,” “Change and Reconstruction,” and “Learning and Absorption,” this paper also follows this classification: First, digital transformation enhances organizational coordination and integration capabilities. It helps enterprises break path dependence, optimize governance structures and processes, and reduce internal costs; simultaneously, it promotes effective management and collaboration of data resources, alleviating information asymmetry, enabling enterprises to respond precisely to stakeholder needs [30,31]. Second, digital transformation improves change and reconstruction capabilities. By introducing advanced IT and data analysis tools, it drives enterprises towards intelligent manufacturing and builds dedicated digital platform capabilities [32]. This innovation capability drives the reallocation of resources, thereby achieving the reconstruction of the value chain [33]. Finally, digital transformation strengthens learning and absorption capabilities. As the foundation of dynamic capabilities, it promotes the generation, transmission, and institutionalization of new knowledge [34]. This not only supports the construction of efficient digital decision-making processes [35] but also drives the organization towards platform-based and agile management transformation, reshaping capability structures and driving business model innovation, significantly enhancing organizational flexibility and response speed in dynamic environments [36,37].

In summary, the improvement of dynamic capabilities enables enterprises to use digital technologies to open up resource circulation channels, precisely match human, financial, and material resources, reduce misallocation and idleness, thereby reducing costs and improving production efficiency. Based on this, we propose the hypotheses:

- H2: Digital transformation enhances enterprise Total Factor Productivity by improving dynamic capabilities.
- H2a: Digital transformation enhances enterprise Total Factor Productivity by improving organizational coordination and integration capabilities.
- H2b: Digital transformation enhances enterprise Total Factor Productivity by improving change and reconstruction capabilities.
- H2c: Digital transformation enhances enterprise Total Factor Productivity by improving learning and absorption capabilities.

## Research design

### Data sources

This paper uses A-share listed companies from 2010 to 2023 as the sample and performs the following screening and processing on the initial sample:

1. Delete samples where total assets are less than total liabilities;
2. Delete financial enterprises;
3. Delete ST and *ST enterprises;
4. Delete samples with an enterprise age of less than 1 year;
5. Exclude samples with missing key variables and apply winsorization at the 1% level on both sides for continuous variables.

### Construction of digital transformation indicators

**Fig 1:**
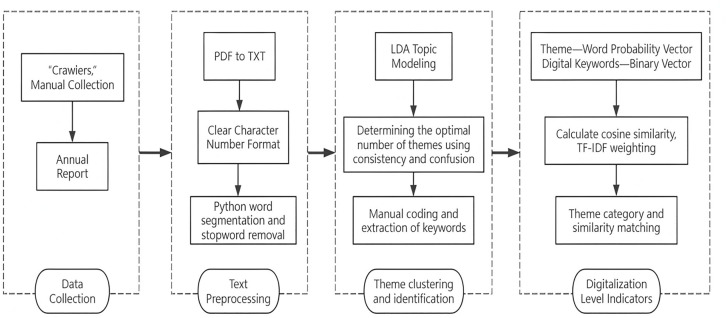
LDA-based digitization level indicator construction process.

Given that traditional dictionary methods struggle to mine deep text semantics and suffer from insufficient measurement validity, this paper employs the LDA (Latent Dirichlet Allocation) topic model to perform natural language processing on the annual report texts of listed companies. We construct a measurement path of “data collection—semantic analysis—element extraction” to accurately depict the “diverse direction” characteristics of enterprise digital transformation. The overall process is shown in Fig 1.

#### Data acquisition and preprocessing.

This study selects the annual reports of A-share non-financial, non-ST listed companies from 2010 to 2023 as the data source (obtained via Cninfo and official company websites, using a combination of Python crawling and manual collection). First, the raw text is deeply cleaned to remove punctuation, headers, numbers, and special characters. Second, a customized stop-word list (e.g., “Board of Directors,” “Company”) is used for noise reduction, and the Jieba segmentation tool is employed to build a standardized corpus.

#### LDA topic model training and identification.

**Fig 2:**
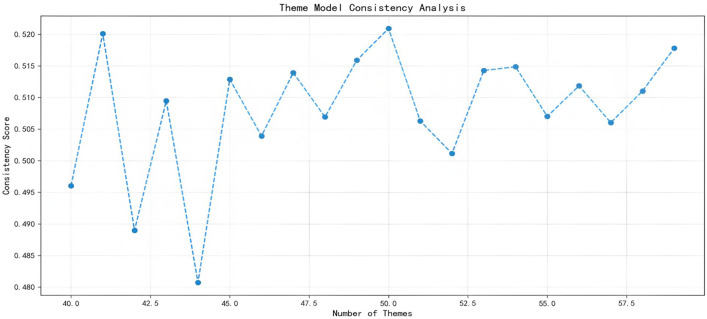
Corporate Annual Reports – Change in Number of Themes.

The Gensim package is used to build the LDA topic model, with Topic Coherence Score as the optimization criterion [38]. By traversing different numbers of topics and evaluating their coherence scores, the optimal number of topics is determined to be 50 (as shown in Fig 2). Subsequently, the 50 initial topics identified are manually semantically encoded to extract the Top-N keywords and their probability distributions for each topic.

#### Topic mapping mechanism based on “TF-IDF weighted cosine similarity”.

To accurately classify the 50 initial LDA topics into specific digital technology directions, this paper constructs a mapping mechanism based on the Vector Space Model, with the following steps:

**Step 1: Construction of Direction Feature Vectors (Seed Lexicon Vectorization).** Drawing on the research of [10] and [23], this paper deconstructs digital transformation into five specific directions: Artificial Intelligence, Big Data, Cloud Computing, Blockchain, and Digital Technology Application. For each direction, a “seed lexicon” comprising high-frequency technical terms is compiled. The TF-IDF (Term Frequency-Inverse Document Frequency) algorithm is then employed to transform the seed lexicon of each direction into a feature vector, $V_{dir_{j}}$(*j* = 1,...,5), within a high-dimensional space. This vector not only accounts for the frequency of keyword occurrences but also reduces the weight of generic terms through inverse document frequency, thereby highlighting the unique technical semantics inherent to each direction.

**Step 2: Topic Vectorization and Similarity Calculation.** For the 50 initial topics identified by the LDA model, the Top-N keywords and their respective probability distributions are extracted for each topic. Similarly, TF-IDF weighting is applied to generate topic feature vectors, $V_{topic_{k}}$(*k* = 1,...,50). Subsequently, the Cosine Similarity algorithm is utilized to calculate the cosine of the angle between each topic vector and the five direction feature vectors, serving as a measure of semantic proximity. The calculation formula is as follows:


$$S_{k,j}=\cos(\theta )=\frac{V_{{\text{topic}}_{k}}\cdot V_{{\text{dir}}_{j}}}{\left\|V_{{\text{topic}}_{k}}\right\|\times \left\|V_{{\text{dir}}_{j}}\right\|}=\frac{\sum_{i=1}^{n}w_{k,i}\times w_{j,i}}{\sqrt{\sum_{i=1}^{n}w_{k,i}^{2}}\times \sqrt{\sum_{i=1}^{n}w_{j,i}^{2}}}$$ (1)

Where *S*_*k*,*j*_ denotes the similarity score between the *k*-th topic and the *j*-th digital direction, with a value range of [0, 1]. A value closer to 1 indicates a higher semantic alignment between the topic and that specific digital direction. This process generates a 50×5 similarity matrix.

**Step 3: Mapping Rules and Threshold Screening.** For the generated 50×5 similarity matrix, this paper implements the following mapping and denoising protocols. It is important to note that rather than relying on subjective “intermediate-level clustering” or simple hard-threshold elimination methods, we adopt a more robust mechanism termed “Maximum Similarity Matching + Sigmoid Weight Decay”:

**Maximum Similarity Matching Principle:** For each LDA topic *k*, we traverse its similarity scores $\left\{S_{k,1},S_{k,2},...,S_{k,5}\right\}$ across the five digital directions and assign it to the direction j^*^ yielding the highest score, defined as:


$$j^{*}=\mathrm{\backslash arg}\max_{j}(S_{k,j})$$ (2)

**Noise Suppression via Sigmoid Function:** Recognizing that certain topics may exhibit low semantic correlation with all digital directions (i.e., “noise topics”), direct weighting could introduce interference. To address this, drawing on the concept of activation functions in neural networks, this paper introduces a Sigmoid transformation to perform non-linear mapping on the original similarity scores, thereby achieving soft denoising. The specific formula is as follows:


$$W_{k,j}=\frac{1}{1+e^{-\alpha \cdot S_{k,j}}}$$ (3)

**Step 4: Indicator Aggregation and Final Calculation Formula.** After determining the directional attribution of each valid topic, we aggregate the digital intensity for enterprises across each direction. For the annual report of enterprise *i* in year *t*, the degree of digital transformation belonging to direction *j*, denoted as Digital_*i*,*t*,*j*_, is defined as the sum of the topic probabilities for all topics assigned to direction *j* within that annual report:


$${\text{Digital}}_{i,t,j}=\sum_{k=1}^{50}\left(P_{i,t,k}\times 𝕀(j_{k}^{*}=j)\times W_{k,j_{k}^{*}}\right)$$ (4)

Where P_*i*,*t*,*k*_ represents the topic distribution probability; $𝕀(j_{k}^{*}=j)$ is an indicator function ensuring that only topics assigned to direction *j* are accumulated; and $W_{k,j_{k}^{*}}$ denotes the weight coefficient adjusted by the Sigmoid function, which automatically filters out noise interference with low relevance.

The advantages of this method are twofold: First, the automated vector matching implemented via code eliminates the subjectivity and arbitrariness inherent in manual coding. Second, by leveraging TF-IDF and Cosine Similarity, the method precisely captures deep semantic associations, thereby ensuring the objectivity, reproducibility, and transparency of the indicator construction.

## Variable definitions

### Dependent variable

This paper sets Enterprise Total Factor Productivity (TFP) as the dependent variable. Referencing the ACF method proposed by [39], enterprise TFP is calculated in the form of value added. Specifically, operating revenue represents total output, cash paid for purchasing goods and accepting services measures intermediate inputs, and capital and labor inputs are measured by net fixed assets and the number of employees, respectively. Value added is calculated as Total Output minus Intermediate Inputs. Additionally, in subsequent sections, the LP method [40] and OP method [41] will be used to recalculate enterprise TFP to replace the dependent variable for robustness checks.

### Independent variables

#### Core independent variable: Level of digital transformation.

The core independent variable of this paper is the level of enterprise digital transformation (Digital). Unlike traditional word frequency statistics, this paper uses the aforementioned LDA topic model to mine deep semantics in annual report texts, subdividing digital transformation into five structurally different technology directions: Artificial Intelligence, Big Data, Cloud Computing, Blockchain, and Digital Technology Application. The specific indicator construction logic, model training process, and algorithmic details are detailed in the section “Construction of digital transformation indicators” above. In empirical analysis, this paper adopts a multi-dimensional variable usage strategy:

- Composite Index (Digital): Summing the five sub-dimension indicators to examine the overall effect of digital transformation.
- Sub-dimension Indicators (AI, BD, CC, BC, DTA): Using the five sub-indicators separately to examine the differentiated impacts of digital “diverse direction” transformation and identify the asymmetric action mechanisms of different technology paths.

#### Theoretical basis and connotation of dimension division.

The division of digital transformation directions in this study is mainly based on the “Technology-Application” dual-layer framework proposed by [10], referencing the expansion and refinement of digital technology keywords by [23] and [11]. Specifically, the four underlying technologies of Artificial Intelligence, Big Data, Cloud Computing, and Blockchain (i.e., the “ABCD” architecture) and the fusion layer of Digital Technology Application together constitute the five digital transformation directions of this paper (see Table 1).

**Table 1:** Business Transformation Directions and Theme Words.

Direction	Segmentation Dictionary
Artificial Intelligence	Artificial Intelligence, Business Intelligence, Image Understanding, Investment Decision Support Systems, Intelligent Data Analysis, Intelligent Robots, Machine Learning, Deep Learning, Semantic Search, Biometric Technology, Face Recognition, Voice Recognition, Identity Verification, Autonomous Driving, Natural Language Processing, Automatic Control, Automatic Monitoring, Automatic Surveillance, Automatic Detection, Automatic Production, Numerical Control, Integration, Integrated Solutions, Integrated Control, Integrated Systems, Industrial Cloud, Future Factory, Intelligent Fault Diagnosis, Lifecycle Management, Manufacturing Execution Systems, Virtualization, Virtual Manufacturing
Big Data	Big Data, Data Mining, Text Mining, Data Visualization, Heterogeneous Data, Augmented Reality, Mixed Reality, Virtual Reality
Digital Technology Application	Mobile Internet, Industrial Internet, Mobile Interconnection, Internet Healthcare, E-commerce, Mobile Payment, Third-party Payment, NFC Payment, Smart Energy, B2B, B2C, C2B, C2C, O2O, Networked, Smart Wearables, Smart Agriculture, Smart Transportation, Smart Healthcare, Smart Customer Service, Smart Home, Smart Investment Advisory, Smart Tourism, Smart Environmental Protection, Smart Grid, Smart Marketing, Digital Marketing, Unmanned Retail, Internet Finance, Digital Finance, Fintech, Financial Technology, Quantitative Finance, Open Banking, Data Management, Data Networks, Data Platforms, Data Centers, Data Science, Digital Control, Digital Technology, Digital Communication, Digital Networks, Digital Intelligence, Digital Terminals, Digitization, Big Data, Unmanned Sales, Industrial Internet, Internet Solutions, Internet Technology, Internet Thinking, Internet Action, Internet Business, Internet Mobile, Internet Applications, Internet Marketing, Internet Strategy, Internet Platforms, Internet Models, Internet Business Models, Internet Ecosystem, E-commerce, Internet, Internet+, Online-Offline, Online to Offline, Online and Offline, Information Sharing, Information Management, Information Integration, Information Software, Information Systems, Information Networks, Information Terminals, Information Centers, Informatization, Networking, Industrial Information, Industrial Communication, Cyber-Physical Systems
Cloud Computing	Cloud Computing, Stream Computing, Graph Computing, In-Memory Computing, Secure Multi-Party Computing, Brain-Inspired Computing, Green Computing, Cognitive Computing, Converged Architecture, Hundred-Million-Level Concurrency, EB-Level Storage, Internet of Things, Cloud IT, Cloud Ecosystem, Cloud Services, Cloud Platforms
Blockchain	Blockchain, Digital Currency, Distributed Computing, Differential Privacy Technology, Smart Financial Contracts, Bitcoin, Consensus Mechanisms, Consortium Chains, Decentralization

This five-dimensional classification is adopted primarily based on two considerations. First, the need for policy alignment and practical representativeness. This classification system not only closely echoes the background of the rapid development of the global digital economy but also effectively captures the real digital strategic orientations reflected in formal information disclosures such as corporate annual reports. However, existing research indicates that different digital technologies have significant heterogeneity in their impact on corporate performance [10]; therefore, measuring digital levels solely with a composite index can easily obscure the asymmetry of the action mechanisms of various technology paths, leading research into an “average number trap.” Second, aiming to crack the black box of the “Solow Productivity Paradox.” This paper retains and operationalizes this multi-dimensional structure with the core purpose of identifying the differentiated roles of different technology directions in activating enterprise dynamic capabilities and thereby enhancing TFP. Specifically, Artificial Intelligence may drive innovation more through “intelligent manufacturing integration,” while Big Data focuses on optimizing management efficiency. Thus, this subdivided perspective helps provide finer empirical evidence for understanding the complex economic consequences of digital transformation.

#### Special dimension explanation: Digital technology application.

It needs to be specially explained that regarding the potential issue of broad connotation of the “Digital Technology Application” dimension, this paper argues that this dimension does not point to a specific technology but aims to characterize the comprehensive form of high integration and scenario-based landing of underlying digital technologies in business practice. The richness of its content precisely reflects the breadth and penetration of digital transformation in business model reconstruction, user interaction innovation, and industrial ecosystem reshaping. Including it as an independent dimension helps test the unique contribution of such integrated and platform-based digital applications to enterprise productivity, forming a complementary perspective with the examination of single underlying technologies, jointly constituting a complete understanding of the multi-dimensional effects of digital transformation.

### Mediating variables

As a core organizational routine for enterprises to perceive environmental changes, integrate and reconfigure resources, and continuously create competitive advantages [25], dynamic capability is essentially a high-order, implicit, and highly context-dependent management process. Its intrinsic complexity determines that direct and comprehensive empirical measurement faces significant challenges. Although questionnaire surveys can capture managers’ subjective perceptions of capabilities to some extent, they are difficult to apply to causal inference research with large samples and long panels due to limitations in sample size, common method bias, and data timeliness [42]. Mainstream existing literature generally uses observable objective indicators as proxy variables for dynamic capabilities. While unable to fully restore their procedural essence, they offer important advantages in controlling endogeneity and improving external validity. Therefore, this paper constructs panel data structures and, referencing the research ideas of [43], realizes the measurement of dynamic capabilities based on financial indicators.

Acknowledging the aforementioned methodological limitations, and based on dual considerations of theoretical logic and empirical feasibility, this paper deconstructs dynamic capabilities into three dimensions: Organizational Coordination and Integration Capability, Change and Reconstruction Capability, and Learning and Absorption Capability. Proxy variables with sufficient theoretical support are selected for measurement, with the specific construction logic as follows:

First, *Organizational Coordination and Integration Capability* reflects the ability of an enterprise to efficiently allocate and coordinate internal and external resources to optimize operational efficiency [29]. The effectiveness of this capability is ultimately manifested in the improvement of resource allocation efficiency and the optimization of cost structures. Accordingly, this paper selects *Return on Assets (ROA)* and *Operating Cost Ratio (OCR)* as its proxy variables. Among them, ROA measures the efficiency of an enterprise in using total assets to create net profit, serving as a comprehensive financial representation of resource integration effectiveness; OCR directly reflects the level of internal process coordination and supply chain management, embodying the refinement of operational management. Together, from the two complementary perspectives of “output efficiency” and “cost control,” they constitute effective explicit indicators for this dimension [21,44].

Second, *Change and Reconstruction Capability* emphasizes the initiative of an enterprise to achieve strategic transformation through technological innovation and business model updates [33]. The formation of this capability relies on continuous R&D investment and is ultimately condensed into identifiable knowledge outputs. Therefore, this paper adopts *R&D Expenditure* and *Total Patent Grants* to construct an “Input-Output” dual-dimensional measurement system. R&D expenditure represents the resource commitment and strategic intent of the enterprise in technological change [45], while the number of patent grants is a key marker of the institutionalization and marketization of innovation outcomes [46]. This combination effectively captures the complete chain of change and reconstruction capability from resource input to value realization.

Third, *Learning and Absorption Capability* refers to the potential of an organization to identify, acquire, and digest external knowledge, and apply it to business practices. Its core foundation lies in the reserve of high-quality human capital. Given that the organizational learning process is difficult to observe directly, this paper, drawing on [34] and [47], uses the *Proportion of Employees with Bachelor’s Degree or Above (DH)* as a proxy variable. Highly educated employees usually possess stronger information processing capabilities, cross-domain knowledge integration abilities, and a willingness for continuous learning, serving as key carriers for organizational absorption of external knowledge. Although this indicator focuses on the “potential foundation” of capability rather than the “dynamic process,” under the constraint of lacking micro-behavioral data, as a structural antecedent of learning and absorption capability, it has full theoretical rationality and empirical operability.

In summary, although the dynamic capability measurement system constructed in this paper is an indirect approximation of the theoretical construct, every proxy variable is rooted in clear theoretical logic and widely supported by existing literature. By focusing on observable results after capabilities act on enterprise operations, this scheme effectively realizes the operationalization of the multi-dimensional connotation of dynamic capabilities under the premise of ensuring the feasibility of large-sample empirical research, providing a reliable data foundation for subsequently testing the mechanism path where digital “diverse direction” transformation affects TFP through dynamic capability reconstruction.

### Control variables

Drawing on [11,23], and [48], this paper selects a series of enterprise-level control variables to control the impact of other potential factors on the dependent variable. The specific meanings of relevant variables are shown in Table 2.

**Table 2:** Variable Definitions.

Variable Type	Variable Name	Symbol	Construction Method
Dependent	Enterprise TFP	TFP_ACF	Estimated by ACF method
Independent	Digital Transformation	Digital	Estimated by LDA method
Control	Property Rights	Npr	1 for SOEs, 0 for non-SOEs
	Proportion of Independent Directors	Pid	Number of Independent Directors/Board Size
	Ownership Concentration	Share	Sum of shareholding ratios of top 5 shareholders
	Enterprise Age	Age	Natural log of (Observation Year – IPO Year + 1)
	Enterprise Size	Size	Natural log of Total Assets at year-end
	Asset-Liability Ratio	Alr	Total Liabilities / Total Assets
	Return on Equity	Roe	Net Profit / Shareholder Equity Balance
	Current Ratio	Cr	Current Assets / Total Assets
Mediator	Organizational Coordination and Integration Capability	Roa	ROA = Net Profit / Total Assets
	Organizational Coordination and Integration Capability	Ocr	OCR = Operating Cost / Operating Revenue
	Change and Reconstruction Capability	Lnrd	Log of R&D Expenditure
	Change and Reconstruction Capability	Pn	Total Patent Grants
	Learning and Absorption Capability	Dh	Proportion of employees with Bachelor’s degree or above

## Model construction

To empirically test the impact of digital “diverse direction” transformation on enterprise TFP and its mechanism through dynamic capability reconstruction, this paper constructs the following econometric models.

### Baseline regression model

First, to test the direct impact of digital transformation on TFP, this paper constructs a two-way fixed effects model as the baseline regression:


$$TFP_{it}=\alpha +\alpha_{1}Digital_{it}+\sum \alpha_{n}Controls_{it}+\mu_{i}+\gamma_{t}+\varepsilon_{it}$$ (5)

Where the subscripts *i* and *t* denote the enterprise and year, respectively; *TFP*_*it*_ represents the Total Factor Productivity of enterprise *i* in year *t*, estimated using the ACF me*t*hod proposed by [39]; and *Digital*_*it*_ signifies the level of digital transformation for enterprise *i* in year *t*. *Controls*_*it*_ refers to a set of control variables covering dimensions such as enterprise size, age, financial leverage, equity structure, and corpora*t*e governance. Their selection follows common practices in empirical research within this field [10,48] to account for the potential impact of enterprise heterogeneity. Furthermore, $\mu_{i}$ and $\gamma_{t}$ represent enterprise individual fixed effects and year fixed effects, respectively, employed to control for time-invariant enterprise characteristics and macroeconomic cycle shocks; $\varepsilon_{it}$ is the random error term. The estimated coefficient $\alpha_{1}$ in the model measures the marginal impact of digital transformation on enterprise TFP. If $\alpha_{1}$ is significantly positive, it indicates that digital transformation has a significant enhancing effect on enterprise TFP, thereby supporting the research hypothesis of this paper.

### Mediating effect model

To further reveal the internal mechanism by which digital transformation affects TFP, this paper, based on the perspective of dynamic capability reconstruction, adopts the three-step method for testing mediating effects proposed by [49]. Given that the mediating variable in this paper—dynamic capability—includes five specific measurement indicators (i.e., ROA and OCR for organizational coordination and integration capability; LnRD and PN for change and reconstruction capability; DH for learning and absorption capability), to simplify expression and unify the model form, the following uses *MED*_*it*_ to collectively refer to the above five mediating variables. In specific empirical testing, these five indicators will be substituted sequentially for regression analysis. The mediating effect models are set as follows:


$$MED_{it}=\beta_{0}+\beta_{1}Digital_{it}+\sum \beta_{n}Controls_{it}+\mu_{i}+\gamma_{t}+\varepsilon_{it}$$ (6)

$$TFP_{it}=\gamma_{0}+\gamma_{1}Digital_{it}+\gamma_{2}MED_{it}+\sum \gamma_{n}Controls_{it}+\mu_{i}+\gamma_{t}+\varepsilon_{it}$$ (7)

In the equations above, with the exception of the mediating variable *MED*_*it*_, all other variables are defined consistently with the baseline regression model (Eq 1). The coefficient $\beta_{1}$ measures the impact of digital transformation on dynamic capabilities (the mediating variable); $\gamma_{2}$ quantifies the effect of dynamic capabilities on Total Factor Productivity (TFP) after controlling for the level of digital transformation; and $\gamma_{1}$ represents the direct effect of digital transformation on TFP after the inclusion of the mediating variable.

The logic for testing the mediating effect is as follows: First, the coefficient $\alpha_{1}$ in Eq (1) must be statistically significant to confirm the existence of the total effect. Second, both the coefficient $\beta_{1}$ in Eq (2) and the coefficient $\gamma_{2}$ in Eq (3) must be simultaneously significant, indicating that digital transformation can indirectly influence TFP by shaping dynamic capabilities. If, under these conditions, the direct effect coefficient $\gamma_{1}$ in Eq (3) remains significant but its magnitude is smaller than $\alpha_{1}$, it implies that dynamic capabilities play a *partial mediating role*. Conversely, if $\gamma_{1}$ becomes insignificant, it suggests a *complete mediating role*. Through this series of models, this paper aims to open the “black box” regarding how digital transformation affects productivity and to empirically verify whether the reconstruction of dynamic capabilities serves as a critical transmission mechanism between the two.

## Empirical analysis and testing

### Descriptive statistics

Table 3 presents the descriptive statistics of the main variables. The mean of enterprise total factor productivity (*TFP*_*ACF*) is 7.873, ranging from 0 to 12.872, indicating significant TFP differences across enterprises. The mean of digital transformation intensity (*Digital*) is 0.073, with a range of 0.007 to 0.261, suggesting that most enterprises have a low level of digital transformation, while a few achieve high intensity, highlighting the need for further guidance to promote enterprise digital transformation.

**Table 3:** Descriptive Statistics of Main Variables.

Variable Symbol	Obs	Mean	Std. Dev.	Min	Max	VIF
TFP_ACF	28298	7.873	1.657	0	12.872	
Digital	28298	0.073	0.070	0.007	0.261	1.03
Npr	28298	0.367	0.482	0	1	1.16
Pid	28298	37.678	5.653	0	80	1.01
Share	28298	52.046	15.439	2.979	99.23	1.12
Age	28298	2.966	0.328	1.099	4.043	1.09
Size	28298	22.316	1.310	17.654	28.697	1.45
Alr	28298	0.424	0.199	0.008	0.997	1.73
Roe	28298	0.036	0.558	−46.66	23.739	1.03
Cr	28298	2.401	2.978	0.079	144	1.42

### Baseline estimation results of main effects

Table 4 reports the fixed effects regression results of digital transformation on enterprise TFP. This paper adopts a progressive regression strategy: In Column (1), the coefficient of Digital Transformation (Digital) is 0.997, significantly positive at the 1% level; in Column (2), after adding enterprise-level control variables, the coefficient drops to 0.665 but remains highly significant. This result is consistent with the findings of [3] and [48], supporting Hypothesis H1 of this paper, indicating that enterprise digital transformation has a robust promoting effect on TFP. From an economic significance perspective, taking Column (2) as the standard, for every 1 unit increase in the degree of digitization, TFP increases by an average of 0.665; relative to the sample mean of 7.873, this increase is approximately 8.45% (i.e., 0.665/7.873 × 100%), indicating that digital transformation not only has statistical significance but also possesses important economic value.

**Table 4:** Baseline Regression of Main Effects.

VARIABLES	(1) TFP_ACF	(2) TFP_ACF
Digital	0.997***	0.665***
	(0.186)	(0.179)
Npr		−0.0706
		(0.0832)
Age		−0.764***
		(0.199)
Size		0.000517
		(0.0404)
Alr		−1.305***
		(0.154)
Roe		0.112***
		(0.0301)
Cr		−0.0106**
		(0.00469)
Share		0.00328*
		(0.00184)
Pid		0.00451*
		(0.00264)
Constant	7.801***	10.34***
	(0.0135)	(1.062)
N	28,298	28,298
R^2^	0.530	0.539
Year	YES	YES
Company	YES	YES

Note: ***, **, and * indicate significance levels of 1%, 5%, and 10%, respectively; the figures in parentheses are robust standard errors at the enterprise level; the same applies to the following tables.

### Testing of “diverse direction” dimensions of digital transformation

To avoid biases caused by differences in sample selection, this paper first includes these five variables simultaneously in a joint regression on the unified full sample to ensure a consistent comparison baseline. Table 5 reports the estimation results of the impact of five digital transformation directions—Artificial Intelligence (AI), Big Data (BD), Digital Technology Application (DTA), Cloud Computing (CC), and Blockchain (BC)—on enterprise TFP (TFP_ACF). On this basis, a Wald coefficient constraint test (test command) is formally used to examine the statistical significance of the differences in the impact of various digital transformation paths on enterprise productivity.

**Table 5:** Testing of Five Dimensions of Digital Transformation.

VARIABLES	(1)	(2)	(3)	(4)	(5)
AI	0.222***				
	(0.0763)				
BD		0.272**			
		(0.108)			
DTA			0.188***		
			(0.0717)		
CC				−0.238	
				(0.148)	
BC					0.170
					(0.200)
Controls	YES	YES	YES	YES	YES
Constant	10.48***	10.48***	10.48***	10.48***	10.48***
	(1.066)	(1.066)	(1.066)	(1.066)	(1.066)
N	28,298	28,298	28,298	28,298	28,298
R^2^	0.539	0.539	0.539	0.539	0.539
Year	YES	YES	YES	YES	YES
Company	YES	YES	YES	YES	YES

The results show that the promoting effect of Artificial Intelligence (AI) is the most significant, with a coefficient of 0.222 (*p* < 0.01), indicating that enterprise investment in the field of AI has a strong positive driving effect on improving productivity; Big Data (BD) and Digital Technology Application (DTA) also show robust positive effects, with coefficients of 0.272 (*p* < 0.05) and 0.188 (*p* < 0.01) respectively, indicating that data-driven innovation and technology integration are important pathways for efficiency improvement. However, the coefficient for Cloud Computing (CC) is −0.238 (*p* > 0.1) and fails the significance test, implying that at the current stage, the application of cloud computing may not yet bring productivity improvements, or there exists a “digital divide” effect—i.e., enterprises have only achieved infrastructure upgrades but have not yet completed business process reconstruction and value conversion. The coefficient for Blockchain (BC) is 0.170 (*p* > 0.1), also insignificant, possibly because this technology is still in an early exploration stage, and its economic value has not been fully released.

More importantly, the joint test result of these five coefficients is highly significant (*F*(4, 3050)=2.67, *p* = 0.0304), indicating that there are significant differences in the impact of different digital transformation paths on enterprise TFP. This finding supports the necessity of subdivided analysis by technology type and shows that the economic returns of digital transformation have obvious technological heterogeneity. Hypothesis H1a is established.

This finding deepens and refines the conclusions of [2] and [3] regarding the overall positive effect of digital transformation, revealing the heterogeneous characteristics of digital technology impacts. Particularly, the insignificant results of Cloud Computing and Blockchain provide new micro-evidence for explaining the ‘Solow Productivity Paradox’, which is consistent with the views of [8] and [50], i.e., there is a long ‘time lag’ from the deployment of general-purpose technologies to the generation of economic benefits, and enterprises may still be in the ‘installation period’ rather than the ‘harvest period’. In addition, this also confirms the findings of [20], i.e., the performance manifestation of emerging technologies such as blockchain highly depends on the coordination of specific governance structures, warning us not to simply equate all digital technologies.

### Robustness checks

#### Instrumental variable method.

In constructing the instrumental variables for this study, we followed the approach of [23] and [51] by using the number of employed persons (in 10,000 persons) in urban units in the information transmission, software, and information technology services industries of the province where the enterprise is located for verification. The instrumental variable test results show that the F-statistic is 56.988, which is significantly greater than the critical values at the 10% and 15% levels, indicating the absence of weak instruments; the Kleibergen-Paap rk LM statistic is 15.890 (*p* = 0.0001), significantly rejecting the null hypothesis of underidentification. Table 6 reports the regression results of the instrumental variables. The first-stage regression results show that the coefficient of the instrumental variable is significantly positive, indicating that the instrumental variable is significantly positively correlated with enterprise digital transformation; the second-stage regression results show that the coefficient of digital transformation is significantly positive, indicating that after using instrumental variables to weaken the impact of endogeneity problems, the research conclusion still holds.

**Table 6:** Heckman Two-Stage Regression Method.

VARIABLES	(1) Digital	(2) TFP_ACF
Digital		11.67**
		(5.284)
IV	−0.000299***	
	(7.37e-05)	
Npr	−0.00553*	0.00521
	(0.00321)	(0.0874)
Age	−0.0468***	−0.190
	(0.0109)	(0.340)
Size	0.00140	−0.0219
	(0.00138)	(0.0386)
Alr	−0.0203***	−0.864***
	(0.00515)	(0.194)
Roe	0.000570	0.111***
	(0.000495)	(0.0308)
Cr	0.000546**	−0.0132**
	(0.000270)	(0.00616)
Share	0.000614***	−0.00521
	(8.11e-05)	(0.00390)
Pid	8.30e-05	0.00332
	(0.000104)	(0.00250)
Constant	0.163***	
	(0.0425)	
Year	YES	YES
Company	YES	YES
N	27,689	27,689
R^2^	0.578	−0.270

#### Heckman two-stage regression.

Considering that sample selection bias may also affect estimation results, this paper further uses the Heckman two-step method to correct possible bias issues. Drawing on [52], iin the first stage, we take whether a firm undertakes digital transformation as the dependent variable (1 for transformation, 0 otherwise), control for firm characteristics such as age, size, leverage, and estimate the Inverse Mills Ratio (IMR) via Probit regression. In the second stage, we include the IMR in the regression model. The results of the Heckman second stage in Column (2) of Table 7 show that after correcting for self-selection bias, the main conclusions of this paper remain valid.

**Table 7:** Instrumental Variable Method.

VARIABLES	(1) Ifdit	(2) TFP_ACF
Digital_a		0.411**
		(0.201)
IMR		−0.252***
		(0.0929)
Controls	YES	YES
Constant	−4.101***	12.07***
	(1.098)	(1.243)
Year	YES	YES
Company	YES	YES
N	17,942	17,942
R^2^		0.516

#### Lagging of explanatory variables.

First, considering that the impact of enterprise digital transformation may have a time lag [53], this study lags the level of digital transformation by one period and two periods respectively for regression testing. As shown in Columns (1) and (2) of Table 8, the coefficient estimates for Lag One Period (*l*_*Digital*) and Lag Two Periods (*l*2_*Digital*) are 0.362 (*p* < 0.05) and 0.265 (*p* < 0.1) respectively, both remaining statistically significant, indicating that the promoting effect of digital transformation on enterprise TFP remains significantly positive.

**Table 8:** Results of Lagged Explanatory Variables and PSM Regression.

VARIABLES	(1) TFP_ACF	(2) TFP_ACF	(3) TFP_ACF
Digital			0.654***
			(0.179)
l_Digital	0.362**		
	(0.157)		
l2_Digital		0.265*	
		(0.160)	
Controls	YES	YES	YES
Constant	11.03***	11.76***	10.40***
	(1.130)	(1.147)	(1.068)
Year	YES	YES	YES
Company	YES	YES	YES
N	24,356	21,549	28,267
R^2^	0.581	0.590	0.538

#### Propensity Score Matching (PSM).

Second, considering the differences in initial conditions and self-selection problems in enterprise digital transformation, to alleviate omitted variable and sample selection biases, this study adopts the Propensity Score Matching (PSM) method. Using a digital transformation level of 0.1 as the boundary, the sample is divided into a treatment group and a control group. 1:1 nearest neighbor matching is performed based on enterprise characteristics such as age and size to ensure comparability of company characteristics between groups.

The PSM regression results, as shown in Column (3) of Table 8, display that digital transformation has a significant positive impact on enterprise TFP at the 1% level, consistent with the baseline regression and lag analysis results, further supporting the robustness of the research conclusions.

#### Replacing the dependent variable.

To test the robustness of the baseline regression results to TFP calculation methods, referencing studies by [23] and [54], the LP method and OP method are used to recalculate TFP. The results in Columns (1) and (2) of Table 9 show that the Digital coefficients are both significantly positive.

**Table 9:** Replacing Dependent and Explanatory Variables.

VARIABLES	(1) TFP_LP	(2) TFP_OP	(3) TFP_ACF	(4) TFP_ACF
Digital	0.114**	0.115**		
	(0.0568)	(0.0545)		
Digital_1			1.247**	
			(0.616)	
Digital_2				0.0336***
				(0.0124)
Controls	YES	YES	YES	YES
Constant	−4.821***	−2.980***	10.93***	10.94***
	(0.341)	(0.332)	(0.971)	(0.967)
Year	YES	YES	YES	YES
Company	YES	YES	YES	YES
N	27,813	27,813	27,813	27,813
R^2^	0.924	0.892	0.545	0.545

#### Replacing the explanatory variable.

To avoid measurement errors in the core explanatory variable, further referencing [3] and [10], the digital transformation indicators are reconstructed from four dimensions (*Digital*_1) and five dimensions (*Digital_*2). The results in Columns (3) and (4) of Table 9 show that the positive effect still holds significantly.

The consistent results of the above “Replacing the Dependent Variable” and “Replacing the Explanatory Variable” tests indicate that the promoting effect of digital transformation on enterprise TFP is highly robust and does not depend on specific measurement methods.

### Impact mechanism testing

Existing studies mainly measure dynamic capabilities via questionnaire cross-sectional data, which cannot show their evolution [42]. Thus, following [43], this paper uses panel data and financial indicators to measure dynamic capabilities as follows.

*Organizational Coordination and Integration Capability*. Referencing [29] and [44], Return on Assets (ROA) and Operating Cost Ratio (OCR) are selected as proxy variables for resource integration management capability. The test results in Table 10 show: First, digital transformation significantly improves enterprise ROA and significantly reduces OCR, indicating its preliminary effect of optimizing internal resource allocation. Further mediating effect tests show that after introducing Digital and ROA simultaneously in the regression, the coefficient of ROA is significantly positive; similarly, after introducing OCR, its coefficient is significantly negative. These results satisfy the identification conditions for mediating effects, confirming that digital transformation enhances organizational coordination and integration by increasing ROA and reducing OCR, which partially mediates its positive effect on TFP, thus supporting Hypothesis H2a.

**Table 10:** Impact Mechanism Test of Digital Transformation on Enterprise TFP.

VARIABLES	Organizational Coordination and Integration Capability			
	(1) ROA	(2) TFP_ACF	(3) OCR	(4) TFP_ACF
Digital	0.0191**	0.604***	−0.0694***	0.490***
	(0.00849)	(0.175)	(0.0147)	(0.171)
ROA		3.193***		
		(0.215)		
OCR				−2.527***
				(0.214)
Controls	YES	YES	YES	YES
Constant	−0.248***	11.13***	0.924***	12.68***
	(0.0449)	(1.038)	(0.0808)	(1.059)
Year	YES	YES	YES	YES
Company	YES	YES	YES	YES
N	28,298	28,298	28,298	28,298
R^2^	0.515	0.550	0.838	0.550

*Change and Reconstruction Capability*. This study conducts tests from two dimensions: innovation input and output. The logarithm of R&D expenditure (LNRD) and total patent grants (PN) are used as proxy variables for enterprise R&D capability and technological innovation level, respectively. As shown in Table 11, the coefficient of digital transformation on LNRD is 1.384 (*p* < 0.01), and the coefficient on PN is 0.279 (*p* < 0.05), both of which are significantly *p*ositive. This indicates that digital transformation can directly enhance firms’ R&D investment and innovation out*p*ut. Further mediating effect tests show that after adding LNRD and PN to the model, their coefficients on TFP_ACF are 0.0125 and 0.0258 respectively, and the coefficient of Digital remains significant. This confirms that digital transformation effectively strengthens enterprise change and reconstruction capabilities by promoting R&D investment and improving innovation output, thereby driving productivity improvement, verifying hypothesis H2b.

**Table 11:** Impact Mechanism Test of Digital Transformation on Enterprise TFP.

VARIABLES	Change and Reconstruction Capability				Learning and Absorption Capability	
	(1) LNRD	(2) TFP_ACF	(3) PN	(4) TFP_ACF	(5) DH	(6) TFP_ACF
Digital	1.384***	0.647***	0.279**	0.658***	3.631**	0.641***
	(0.499)	(0.179)	(0.135)	(0.179)	(1.417)	(0.179)
LNRD		0.0125**				
		(0.00548)				
PN				0.0258**		
				(0.0058)		
Controls	YES	YES	YES	YES	YES	YES
Constant	5.892***	10.29***	0.843***	10.79***	3.631**	10.64***
	(0.146)	(1.035)	(0.152)	(1.048)	(1.417)	(1.052)
Year	YES	YES	YES	YES	YES	YES
Company	YES	YES	YES	YES	YES	YES
N	28,298	28,298	28,298	28,298	28,298	28,298
R^2^	0.612	0.551	0.587	0.549	0.539	0.550

*Learning and Absorption Capability*. Referencing [47], this study uses the proportion of employees with a bachelor’s degree or above (DH) as a core indicator of enterprise knowledge reserves and learning conversion potential. This indicator first reflects the proportion characteristics of the highly educated group in the enterprise’s human resource structure, and its core points to the organization’s potential knowledge reserve level and learning conversion capability. From the connotation of learning and absorption capability, this indicator indirectly reflects the enterprise’s efficiency in acquiring new knowledge and technologies, the depth of understanding, and the potential for application: a high proportion usually means the organization possesses sufficient knowledge carriers, making it easier to complete the internal transmission, integration, and institutionalization of knowledge, thereby providing a human resource foundation and execution guarantee for the exertion of learning and absorption capability in dynamic capabilities. On this basis, the knowledge formed through learning and absorption can be effectively transformed and applied to business practices, and the powerful knowledge chain built through continuous learning may also bring competitive advantages to the enterprise [29]. As shown in Table 11, the coefficient of digital transformation (Digital) on DH is 3.631 (*p* < 0.05), which is significantly positive. This suggests that digital transformation significantly optimizes enterprises’ human capital structure and improves the organizational potential foundation for knowledge acquisition and absorption. In the mediating test, the coefficient of DH on TFP_ACF is 0.641 (*p* < 0.01), and the coefficient of Digital remains significant, indicating that learning and absorption capability plays a mediating role between digital transformation and productivity improvement. Therefore, digital transformation promotes the improvement of TFP by expanding high-knowledge human resources and enhancing organizational learning and absorption capability, thus establishing Hypothesis H2c.

In summary, Hypothesis H2 is established. This result supports the views of [4] and [29] regarding resource integration and innovation chain drivers. More importantly, unlike studies such as [42] that mostly use questionnaire surveys, the empirical results of this paper based on objective financial indicators overcome common method bias and more rigorously confirm the “Digital Transformation—Dynamic Capability—Performance” chain proposed by [44].

### Heterogeneity analysis

Given the significant differences among enterprises in terms of factor endowments, industry attributes, and production characteristics, the enabling effect of digital transformation on total factor productivity (TFP) may be asymmetric. To further investigate this heterogeneity, this paper conducts heterogeneity tests from two dimensions: enterprise factor intensity characteristics and industry classification, with the results presented in Table 12.

**Table 12:** Heterogeneity Analysis by Property Rights.

VARIABLES	(1) Labor-Intensive	(2) Capital-Intensive	(3) Technology-Intensive	(4) Manufacturing	(5) Services
Digital	0.800*	−0.00454	0.505***	0.560***	1.014
	(0.453)	(0.429)	(0.171)	(0.184)	(0.689)
Controls	YES	YES	YES	YES	YES
Constant	6.122**	17.29***	10.67***	11.43***	8.801***
	(2.813)	(2.416)	(1.290)	(1.073)	(3.219)
Year	YES	YES	YES	YES	YES
Company	YES	YES	YES	YES	YES
N	8,666	5,003	12,856	19,568	5,993
R^2^	0.578	0.533	0.516	0.528	0.577

#### Heterogeneity analysis based on enterprise factor intensity.

Following the 2012 industry classification standards issued by the China Securities Regulatory Commission (CSRC) and drawing on existing literature, this study categorizes the sample firms into three groups: labor-intensive, capital-intensive, and technology-intensive. This classification aims to examine the heterogeneity in the marginal effects of digital transformation across different factor endowment structures.

The regression results presented in Columns (1) through (3) of Table 12 reveal heterogeneous effects of digital transformation across different factor structures. First, for labor-intensive firms, the coefficient is 0.800 and significant at the 10% level, suggesting that digital technologies effectively substitute for repetitive manual labor and optimize workforce allocation, thereby unleashing significant productivity dividends. Second, for technology-intensive firms, the coefficient stands at 0.505 with significance at the 1% level—higher than that of labor-intensive firms. This robust effect likely stems from these firms’ strong R&D foundations and technological absorption capabilities, which facilitate the efficient integration of digital tools into core business processes, driving greater productivity gains through innovation and knowledge spillovers. In contrast, for capital-intensive firms, the coefficient is −0.00454 and statistically insignificant. This null result may be attributed to the substantial specific assets held by such firms; digital transformation often requires reconstructing underlying infrastructure, entailing high sunk costs and prolonged adjustment periods that obscure short-term productivity improvements.

#### Heterogeneity analysis based on industry attributes.

To further clarify the performance of digital transformation across different industrial sectors, this study stratifies the sample into manufacturing and service industries for grouped regression analysis. As the foundation of the real economy, manufacturing differs fundamentally in its digitalization path from the service sector, which stands as the most active domain for digital economy penetration.

The estimation results in Columns (4) and (5) of Table 12 indicate that for manufacturing firms, the coefficient of digital transformation is 0.560, positive and significant at the 1% level. This validates the pivotal role of digital technologies in the “intelligent-manufacturing integration” process. Through the application of industrial internet and smart manufacturing technologies, manufacturing firms have achieved refined control over production processes and efficient supply chain coordination, significantly enhancing total factor productivity (TFP). Conversely, for service firms, although the coefficient is larger at 1.014, it fails to pass statistical significance tests. This outcome may stem from the complex internal structure of the service sector: on one hand, some modern service industries are already highly digitalized, leading to diminishing marginal gains; on the other hand, digital transformation in traditional consumer services often focuses on front-end marketing rather than back-end efficiency improvements. Furthermore, unlike manufacturing, service output is difficult to measure precisely using standardized metrics, contributing to the lack of statistical significance.

In summary, the dividends of digital transformation are not “universal” but are highly contingent upon a firm’s factor endowment structure and industry attributes. Technology-intensive and labor-intensive firms derive greater benefits from transformation—the former through technological complementarity and the latter through factor substitution—whereas capital-intensive firms, constrained by asset rigidity, exhibit insignificant short-term effects. At the industry level, manufacturing serves as the primary arena where digital transformation boosts TFP, demonstrating the most significant and robust outcomes. These findings suggest that policymakers and corporate managers should avoid a “one-size-fits-all” approach when advancing digital transformation. Instead, they must formulate differentiated strategies tailored to the specific characteristics of various industries and enterprises.

## Conclusion and implications

Based on the perspective of dynamic capability reconstruction, this paper deeply explores the impact of digital “diverse direction” transformation on enterprise Total Factor Productivity (TFP). By constructing the theoretical logic of “Digital ‘Diverse Direction’ Transformation—Dynamic Capability—Total Factor Productivity” and employing the LDA topic model and various econometric methods, an empirical test was conducted on over 3,000 A-share listed companies in China from 2010 to 2023. This clarified the effect characteristics, mechanism paths, and heterogeneity of the transformation, providing a new perspective for cracking the “productivity paradox.” The research conclusions show: First, digital transformation overall significantly promotes TFP, but there is technological heterogeneity. The promoting effect of Artificial Intelligence is the most significant, the effects of Big Data and Digital Technology Application are robust, while Cloud Computing, due to not yet completing process reconstruction, and Blockchain, being in an early exploration stage, both have insignificant impacts, which remains true after robustness checks. Second, the three dimensions of dynamic capability play a key mediating role: digital transformation enhances organizational coordination and integration capabilities by improving ROA and reducing costs; strengthens change and reconstruction capabilities by promoting R&D and innovation; and improves learning and absorption capabilities by optimizing human capital structure, thereby opening the “black box” of impacting productivity. Third, there is significant industry heterogeneity. Digital transformation demonstrates a robust positive effect on total factor productivity (TFP) in the manufacturing sector, validating the potential of “digital-real integration.” In contrast, while the coefficient for the service sector is positive, it fails to achieve statistical significance. This discrepancy primarily stems from the complex structure of the service industry, the difficulty in standardizing output measurements, and diminishing marginal returns in certain sub-sectors, which collectively obscure the overall statistical effect. Consequently, the results exhibit a pattern characterized by “strong manufacturing, weak services.”

This study breaks through the limitations of previous “holistic” analyses by achieving precise segmentation and quantification of technological directions. It explains the “productivity paradox” from the dual perspectives of technological heterogeneity and time lags, while extending the application of Dynamic Capabilities Theory within the digital economy. In terms of practical implications, the research provides clear guidance for both enterprises and governments: firms should adopt differentiated technology strategies—prioritizing AI and big data for cost reduction and efficiency gains, planning cloud computing deployments, and making long-term investments in blockchain. They must also systematically enhance dynamic capabilities by tailoring specific dimensions to target technologies and precisely position themselves based on industry characteristics; specifically, labor-intensive firms should focus on automation to optimize workforce allocation, while capital- and technology-intensive firms need to address asset restructuring pressures and deepen technological integration, respectively, to jointly build a robust industrial ecosystem. Furthermore, establishing mechanisms for tracking transformation outcomes and issuing risk warnings, strengthening digital skills training, and avoiding resource misallocation are crucial to effectively driving the transition of digital transformation from mere “technological investment” to substantive “efficiency output.”

From the perspective of the uniqueness of research contributions, this study responds to the explicit research needs of the existing academic community. [11] and [23] pointed out that the measurement of digital transformation needs to break through the limitations of word frequency statistics and pay attention to the heterogeneity of technology directions; [10] and [48] called for in-depth deconstruction of the differentiated action mechanisms of digital transformation to reasonably explain the “productivity paradox.” This paper conducts research targeting these research gaps generally concerned by the academic community. The proposed measurement methods and research conclusions have clear marginal contributions, making up for the deficiencies in existing literature and possessing significant publication value and academic reference significance.
